# IKBKE regulates renal cell carcinoma progression and sunitinib resistance through the RRM2-AKT pathway

**DOI:** 10.7150/ijbs.102666

**Published:** 2024-11-11

**Authors:** Shiwei Liu, Junhong Li, Junyu Zhang, Fangning Wan, Zongyuan Hong, Zhe Hong, Bo Dai

**Affiliations:** 1 Department of Urology, Fudan University Shanghai Cancer Center, Shanghai, 200032, China.; 2 Department of Oncology, Shanghai Medical College, Fudan University, Shanghai, 200032, China.; 3 Shanghai Genitourinary Cancer Institute, Shanghai, 200032, China.; 4 Laboratory of Quantitative Pharmacology, Wannan Medical College, Wuhu, 241002, China.

**Keywords:** renal cell carcinoma, IKBKE, RRM2, sunitinib, drug resistance

## Abstract

Tyrosine kinase inhibitors (TKIs), such as sunitinib, have emerged as promising agents in renal cell carcinoma (RCC) treatment, particularly in patients at advanced/metastatic clinical stages. However, acquired resistance to sunitinib is common following prolonged clinical treatment in RCC. Increasing evidence has demonstrated a strong correlation between inhibitor of nuclear factor kappa B kinase subunit epsilon (IKBKE) and cancer progression as well as drug resistance. Here, we found that IKBKE is upregulated in RCC tissues and sunitinib-resistant RCC cells. High IKBKE expression is positively correlated with advanced clinical staging and a poor prognosis in RCC. Silencing IKBKE downregulates ribonucleotide reductase M2 (RRM2) and induces cell cycle arrest at G2/M phase, suppressing RCC progression and enhancing sunitinib sensitivity to RCC cells. Mechanistically, IKBKE interacts with and phosphorylates RRM2 to activate the AKT signaling pathway to promotes RCC progression and sunitinib resistance. Notably, the IKBKE inhibitor CYT387 restores sunitinib sensitivity in RCC cells by downregulating RRM2 expression. Collectively, these results indicate that inhibition of IKBKE restrains RCC progression and enhances sunitinib sensitivity by downregulating RRM2 through the RRM2-AKT pathway, suggesting that IKBKE may be a potential therapeutic target for RCC.

## Introduction

Renal cell carcinoma (RCC), a prevalent malignant neoplasm in the urinary system, is estimated to account for 81,180 new cancer cases and 14,890 cancer-relevant deaths in the United States in 2023 1. The prognosis of RCC depends largely on the clinical staging, and patients with advanced stage disease have a markedly poorer prognosis 2. Due to the nonspecific symptoms and features of RCC, many patients are diagnosed late in the disease, resulting in ineffective clinical treatment 3. Incontrovertibly, it is urgent to identify early diagnostic markers for RCC and to explore promising targets for advanced RCC treatment.

To date, systemic interventions have primarily involved antiangiogenesis treatment or immunotherapy, both of which are crucial options for advanced RCC 4. Tyrosine kinase inhibitors (TKIs), such as sunitinib, serve as multitarget antiangiogenic agents and have exhibited great promise in the therapy of advanced/metastatic tumors by targeting vascular endothelial growth factor (VEGF) 5, 6. However, clinical data has indicated that a substantial number of RCC patients exhibit primary resistance to sunitinib or develop acquired resistance to sunitinib during therapy 7. Thus, understanding the molecular biological process underlying TKI resistance is of great importance to establish more efficacious and lasting therapeutic strategies for advanced RCC patients. Inhibitor of nuclear factor kappa B kinase subunit epsilon (IKBKE) is a noncanonical I-kappa-B kinase. Growing evidence indicates that the upregulation of IKBKE is closely related to the oncogenesis and progression of various tumors, including breast cancer 8, prostate cancer 9, and glioblastoma 10. Nevertheless, the specific role of IKBKE in RCC and its underlying mechanism remain enigmatic.

In this study, we demonstrated that IKBKE was upregulated in RCC, and its high expression was correlated with advanced tumor staging and poor prognosis. In addition, we observed that IKBKE was upregulated in samples from sunitinib-resistant patients. Activation of the protein kinase B (AKT signaling pathway has been shown to facilitate cancer cell metastasis and angiogenesis, playing an important role in sunitinib resistance 11, 12. We have identified that IKBKE can regulate sunitinib sensitivity through the AKT signaling pathway by influencing RRM2 expression, which is a crucial component of the AKT signaling pathway. Furthermore, our study demonstrated that the IKBKE inhibitor CYT387 could restrain the proliferation of RCC cells and sensitize RCC cells to sunitinib treatment. Collectively, we identified a potential IKBKE-RRM2 axis that may serve as a promising therapeutic target for RCC.

## Methods and Materials

### Acquisition of raw data and bioinformatics analysis

Through the “TIMER” website (https://cistrome.shinyapps.io/timer/), the mRNA expression of IKBKE in various cancer types was assessed. Moreover, mRNA expression profiling and clinical data were extracted from publicly available databases, specifically The Cancer Genome Atlas (TCGA) (http://cancergenome.nih.gov/) and Gene Expression Omnibus (GEO) (https://www.ncbi.nlm.nih.gov/geo/). As previously described, these data were further subjected to subsequent processing and normalization procedures using “R” software 13. Correlation analyses between IKBKE expression, normal and tumor tissues, and clinical staging were subsequently conducted and compared using the Kruskal-Wallis and Wilcoxon rank sum tests. Furthermore, TCGA-kidney renal clear cell carcinoma (KIRC) patients were grouped into low- and high-IKBKE expression subgroups according to the median IKBKE expression level, and a Kaplan-Meier curve of overall survival (OS) was subsequently constructed using the log-rank test. Moreover, a multivariate Cox regression analysis was performed to assess whether IKBKE expression could serve as an independent prognostic indicator when combined with clinical variables, including age, sex, grade, and stage. In addition, the Gene Set Enrichment Analysis (GSEA) tool (version 4.3.2) (c2.cp.kegg.v7.4.symbols) was employed to investigate potential IKBKE-involved signaling pathways. Through the “STRING” website (https://cn.string-db.org/), a protein-protein interaction (PPI) network encompassing IKBKE and RRM2 along with 55 differentially expressed genes (DEGs) related to sunitinib resistance was established.

### Cell lines and culture

The renal cancer cell lines 769-P, 786-O, and CAKI-1 and the renal tubular epithelial cell line HK-2 were obtained from our laboratory (Shanghai Cancer Center). The 769-P and 786-O cell lines were maintained in RPMI-1640 medium (Gibco, Waltham, MA, USA.), CAKI-1 cells were maintained in MEM (Gibco), and HK-2 cells were cultured in DMEM/F12 medium (Gibco). All culture media were supplemented with 10% FBS (Gibco) and 1% P/S (Gibco) at 5% CO_2_ and 37 °C. An IKBKE inhibitor CYT387 (HY-10961), a sunitinib inhibitor (HY-10255A), a proteasome inhibitor (MG132), and a JAK kinase inhibitor (JAK kinase-IN-1) were employed in the current study, which were purchased from MedChemExpress.

### Plasmids and lentiviral transfection

The following short hairpin RNA (shRNA) sequences were employed: shIKBKE#1: 5′-GCATTGTGCATCGCGACATCA-3′, shIKBKE#2: 5′-TGGGCAGGAGCTAATGTTTCG-3′; shRRM2#1: 5′-GGCTCAGCTTGGTCGACAA-3′, shRRM2#2: 5′-GGAGTGATGTCAAGTCCAA-3′. These sequences were inserted into the pLKO.1 vector (10879; Addgene, Waltham, MA, USA.). IKBKE and RRM2 were incorporated into the pLVX vector (Takara Bio, Beijing, China) using a C-terminal 3 × FLAG or Myc tag. For RNA interference, the pCMV-VSVG plasmid (8454; Addgene) and psPAX2 plasmid (12260; Addgene) in combination with gene-specific shRNA were cotransfected into 293T cells with Lipofectamine 2000 (Thermo Fisher Scientific, Shanghai, China). Following 6 h of incubation, the culture medium was replaced with fresh medium containing 10% FBS. Subsequently, 48 hours after transfection, the medium devoid of 293T cells was harvested and added to the medium containing cultured renal cancer cells. After an additional 48-hour interval, 769-P and 786-O cells infected with lentivirus were subjected to incubation with puromycin (Sigma-Aldrich, 5 µg/mL) to select cells with successful transfection.

### RNA extraction and real-time qPCR

Total RNA was isolated from renal cancer cells using TRIzol (Takara, Japan). Subsequently, reverse transcription was conducted with the extracted RNA (1 µg) and a employing the ReverTra Ace qPCR RT Kit (Toyobo, China). With iQTM SYBR® Green Supermix (Bio-Rad, CA), quantitative real-time polymerase chain reaction (qRT‒PCR) was performed using cDNAs (1 µL) in a final reaction volume of 20 µL. GAPDH was employed to normalize the mRNA expression level of RRM2, and the data are presented as relative fold changes via the 2^-ΔΔCt^ method. The primer sequences designed to target RRM2 were as follows: forward primer: 5′-GTGGAGCGATTTAGCCAAGAA-3′, reverse primer: 5′-CACAAGGCATCGTTTCAATGG-3′.

### Western blotting and immunoprecipitation (IP)

Briefly, cells were lysed with RIPA buffer containing 1/100 PMSF (100 mM), and the protein concentration was quantified using a bicinchoninic acid assay kit. Following the loading and separation of each protein sample with sodium dodecyl sulfate‒polyacrylamide gel electrophoresis on a 10% gel, samples were transferred to polyvinylidene membranes (0.45 µm). Next, the membranes were blocked using 5% nonfat dried milk and subsequently incubated with the appropriate primary and secondary antibodies. The fluorescence intensity of each protein band was quantified using a FluorChem M system (ProteinSimple, Santa Clara, CA, USA.). The primary antibodies utilized in this study included IKBKE (ABclonal, 1:2000), RRM2 (Proteintech, 1:2000), beta-actin (Proteintech, 1:4000), CyclinA (Proteintech, 1:2000), CyclinB1 (Proteintech, 1:2000), AKT (Proteintech, 1:2000), AKT-p(S473) (Proteintech, 1:2000), GSK3β (Proteintech, 1:2000) and GSK3β-p(S9) (Proteintech, 1:2000).

Extracts for IP were prepared with cell lysis buffer for Western blotting and IP blotting (Beyotime, Shanghai, China) containing a protease inhibitor (Topscience, Shanghai, China). The protein A + G beads (Beyotime Biotechnology) or magnetic beads-conjugated mouse anti DDDDK-tag mAb (MCE) were cocultured with proteins on a rotator at 4 °C overnight. The beads were collected and washed with PBS buffer for 6 times. Then the beads were boiled for 10 min and subjected to Western blotting analysis on SDS-PAGE gels. Western blotting was performed as described above.

### Cell proliferation assay

The Cell Counting Kit 8 (CCK-8, Beyotime Biotechnology, Shanghai, China) assay was employed to evaluate cell proliferation. Specifically, 769-P and 786-O cells were seeded into 96-well plates at a density of 1-2 × 10^3^ cells per well. After incubation for various durations (1, 2, 3, 4, and 5 days), 90 μL of serum-free medium supplemented with 10 μL CCK-8 reagent was added to the target wells. Following a 2-h incubation period at 37 °C, a microplate spectrophotometer was utilized to measure the 450 nm OD value in each well.

The EdU assay (Beyotime Biotechnology) was also employed to evaluate cell proliferation. Briefly, cells were incubated with RPMI-1640 medium containing 20 μM EdU for 2 h. Subsequently, the cells were fixed at room temperature with 4% PFA. Afterward, the cells were incubated in PBS containing 0.5% Triton X-100 and exposed to Apollo-Fluor (RiboBio, China) for 30 min in the absence of light, and the cell nuclei were stained with DAPI for 10 min. The percentage of EdU-positive cells was calculated from 500 cells with fluorescence microscopy.

### Colony formation assay

The 769-P and 786-O cells were seeded in 6-well plates at a density of 1000 cells per well and subsequently incubated for a period ranging from 7 to 14 days. The duration was determined by the size of the resulting cell colonies. Subsequently, the cellular colonies were immobilized with 4% PFA for 30 min, and a 0.1% crystal violet stain was applied for 30 min to facilitate visualization and quantification. The diameter of each well in the six-well plate is approximately 35 mm.

### Cell cycle analysis by flow cytometry

The 769-P and 786-O cells were seeded in a 6-well plate (2 × 10^5^ cells/well) and harvested once they reached 70-80% confluence. Following two washes with cold PBS, the cells were incubated in PBS containing propidium iodide (PI, MultiSciences, Hangzhou, China) for 30 min in the dark and then evaluated with a flow cytometer for cell cycle analysis by assessing the PI-stained cells.

### Flow cytometry of Annexin V-PE/7-AAD staining

Briefly, after collecting and double washing the cells with cold PBS, the quantification of apoptotic cells was carried out utilizing an Annexin V-PE/7-AAD apoptosis kit (MultiSciences). The identification and quantification of apoptotic cells was performed by flow cytometry. Samples were analyzed via flow cytometry, where early apoptotic cells (PE^+^ 7-AAD^-^) and late apoptotic cells (PE^+^ 7-AAD^+^) were distinguished based on Annexin V-PE and 7-AAD staining.

### Patient tissues and immunohistochemistry (IHC)

A total of 120 surgical specimens, comprising 60 RCC tissues and 60 corresponding adjacent normal tissues, were procured from RCC patients who had undergone radical nephrectomy or partial nephrectomy. These specimens were subsequently processed to create paraffin-embedded tissue microarrays (TMAs). IHC was performed using a ready-to-use high-potency IHC secondary antibody kit (Absin, Shanghai, China). The corresponding antibodies were employed for IHC: IKBKE (ABclonal, 1:50 dilution) and RRM2 (Proteintech, 1:50 dilution). The tissue sample was assessed based on the staining intensity (negative: 0, weak: 1, moderate: 2, and strong: 3) and the proportion of positively stained cells (0%: 0, 1%-25%: 1, 26%-50%: 2, 51%-75%: 3, and 76%-100%: 4). The final IHC score was calculated as the product of the intensity score and the percentage score. This study was approved by the Ethics Committee (ZL-XP201402), and all patients participating in this study provided informed consent.

### Animal experiments

BALB/C-nu/nu mice, aged 6 weeks and weighing 22-24 grams, were purchased from Jihui Biotech (Shanghai, China) and were individually housed in the Animal Center of Fudan University Shanghai Cancer Center. The mice had free access to food and water, with environmental conditions maintained at a controlled temperature of 22 ± 0.5 °C, humidity of 60% ± 3%, and an automated 12-h light/dark cycle.

After adaptive feeding, all animals were randomly divided into experimental groups for cell injection. Approximately 1 × 10^7^ lentivirus-infected 786-O cells were subcutaneously injected into the mice (n = 6 for each group). When tumor volume reached 100 mm^3^, randomly picked (by using a random number table) tumor-bearing mice were treated with sunitinib (0.2 mg/10g/2d) or CYT387 (1 mg/10g/2d). Tumor dimensions, specifically length (L) and width (W), were measured and recorded using a Vernier caliper every three days, and the tumor volume was calculated utilizing the following formula: 1/2 × L × W^2^. Mice were euthanized at predetermined time points, and the tumor samples were harvested and analyzed in subsequent experiments. The animal experiment protocols were approved by the Institutional Animal Care and Use Committee of Shanghai Veterinary Research Institute (FUSCC-IACUC-2023372).

### Human umbilical vein endothelial cell (HUVEC) tubule formation assay

96-well plates were incubated with 50 μL Matrigel (BD Biosciences, CA, USA) at 37℃ for 30 min and then polymerized. The conditioned cell medium derived from 786-O cells was harvested for the culture of HUVECs. HUVECs (2 × 10^4^) resuspended in 50 μL of the conditioned medium were seeded to 96-well plates and incubated for 12 h. Subsequently, the 96-well plates were imaged with a microscope.

### Statistical analysis

The experimental data were collected from at least three independent experiments except where specified and were expressed as the mean ± standard deviation (SD). Data analysis and graphical representation were conducted with GraphPad Prism 9.0 (San Diego, CA, USA.), R software (version 4.2.1), and Illustrator 2022 (Adobe). The criteria for calculating the experimental results were preestablished. All testing and data analysis were conducted in a blinded manner. The statistical methods included Student's *t* test, one-way analysis of variance (ANOVA), and the Mann-Whitney U test (for nonnormally distributed data between two groups). Relationships between variables were analyzed using Pearson's correlation tests. Survival curves were constructed using Kaplan-Meier survival analysis and compared using the log-rank test. Cox regression models were established for multivariate analysis. Statistical significance was set at p < 0.05.

## Results

### IKBKE is upregulated and correlated with poor survival in RCC

**Figure 1:**
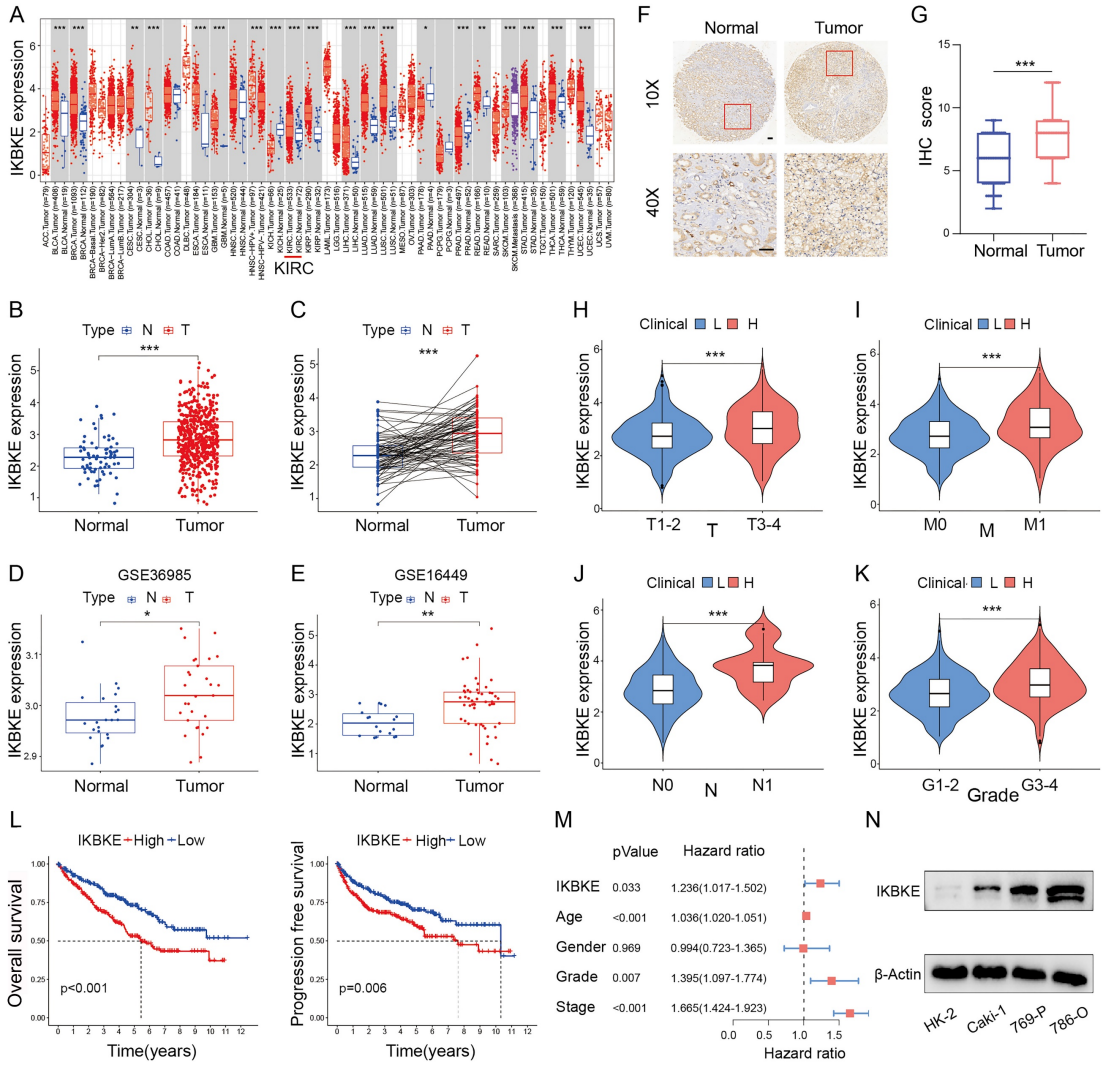
**IKBKE expression is upregulated and associated with poor survival in RCC. (A)** The mRNA expression profile of IKBKE across several cancer types. **(B)** IKBKE mRNA expression in RCC and normal tissues from a TCGA dataset. **(C)** IKBKE mRNA expression in RCC and paired paracancerous tissues from a TCGA dataset.** (D-E)** IKBKE mRNA expression in RCC and normal tissues from GSE36985 and GSE16449. **(F)** Representative immunohistochemical staining of IKBKE in 60 paired RCC samples and normal samples (*n* = 60). Scale bars, 100 μm. **(G)** The IHC scores of IKBKE in normal tissues and RCC tissues (*n* = 60). **(H-K)** Correlations between IKBKE mRNA expression of RCC patients with clinical staging, including T, M, N, and grade. **(L)** The Kaplan-Meier plot of overall survival and progression free-survival for RCC grouped by IKBKE expression. **(M)** Multivariate Cox regression analysis adjusted for IKBKE expression, age, sex, grade, and stage. **(N)** The protein expression levels of IKBKE in RCC and normal kidney cell lines by Western blotting. Each bar represents the mean values ± SD of three independent experiments. Statistical analysis was performed using the Mann-Whitney U test for (A, B, D, E, and H-K), Student's *t* test for (C and G), and log-rank test for (L). *p < 0.05, **p < 0.01, ***p < 0.001.

IKBKE expression across several cancer types was evaluated using the “TIMER” web tool. IKBKE was dramatically upregulated in various types of cancer, including RCC (**Figure 1A**). To verify the upregulation status of IKBKE, we evaluated the mRNA expression of IKBKE in RCC through the TCGA-KIRC, GSE36985 and GSE16449 datasets. Consistently, IKBKE was also found to be upregulated in RCC tissue compared to normal tissue (**Figure 1B-E**). IHC analysis of IKBKE protein expression in RCC patient tissues at our center, Fudan University Shanghai Cancer Center (FUSCC), further supported the elevated expression levels of IKBKE in RCC tissues compared with adjacent benign tissues (**Figure 1F-G**). The correlation of IKBKE expression with clinical tumor staging was evaluated based on the TCGA-KIRC database. Notably, IKBKE expression showed a strong positive correlation with tumor stage, including TNM stage and tumor grade by the Mann-Whitney U test (**Figure 1H-K**). Kaplan-Meier survival analyses suggested that upregulated IKBKE was correlated with poor overall survival (OS) and progression-free survival (PFS) in the TCGA-KIRC dataset (**Figure 1L**). Multivariate Cox regression analysis further emphasized that IKBKE expression could be an independent prognostic factor for RCC (**Figure 1M**). We also assessed IKBKE protein expression in various cell lines, including a renal tubular epithelial cell line (HK-2) and renal cancer cell lines (Caki-1, 769-P and 786-O). We noticed that IKBKE was pervasively overexpressed in RCC cell lines, particularly 786-O cells (**Figure 1N**). Taken together, these results suggest that high IKBKE expression is associated with tumorigenesis and poor prognosis in RCC.

### Oncogenic role of IKBKE in RCC

**Figure 2:**
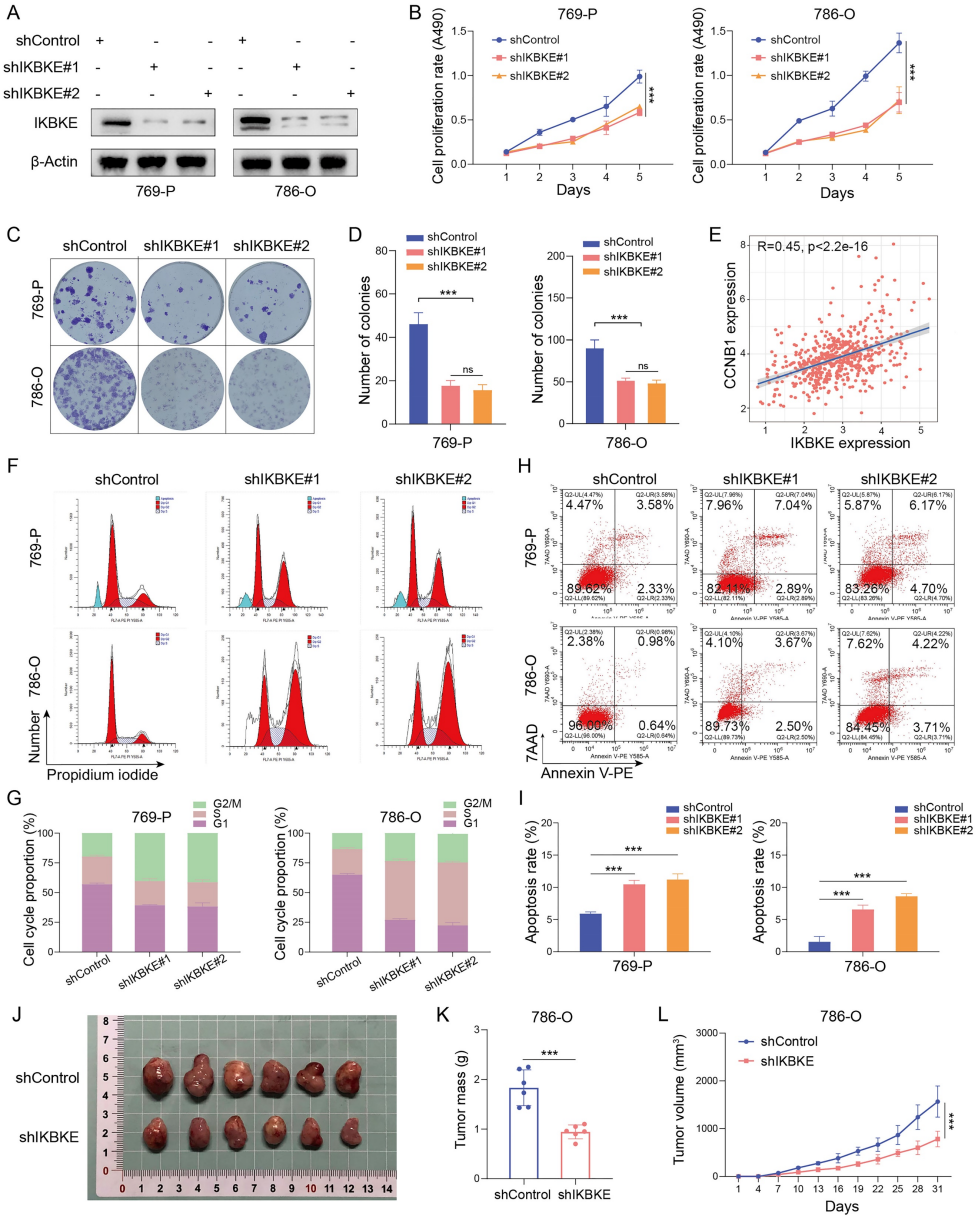
**Oncogenic role of IKBKE in RCC. (A)** Evaluation of IKBKE knockdown efficiency in RCC cell lines through Western blotting. **(B)** The cell proliferation rates in the shControl and shIKBKE groups were assessed in RCC cell lines through a CCK-8 assay. **(C-D)** The colony numbers in the shControl and shIKBKE groups were compared in RCC cell lines through a colony formation assay. The diameter of each well in panel C is approximately 35 mm. **(E)** Correlation between IKBKE mRNA expression and Cyclin B1 mRNA expression.** (F-G)** Cell cycle analysis of RCC cell lines with shControl or shIKBKE through flow cytometry. **(H-I)** The cell apoptosis of RCC cell lines with shControl or shIKBKE through flow cytometry. **(J-L)** 786-O cells stably expressing shControl or shIKBKE-specific shRNAs were injected subcutaneously into nude mice for xenograft assays. The xenografts in each group (*n* = 6 mice per group) were photographed in Panel J, the tumor mass is shown in Panel K, and the tumor growth curve is shown in Panel L. Each bar represents the mean values ± SD of three independent experiments except where stated. Statistical analysis was performed using one-way ANOVA for (B, D, and I), Pearson's correlation analysis for (E), and Student's *t* test for (K and L). ns, no significance, **p < 0.01, ***p < 0.001.

To investigate the potential function of IKBKE in RCC, shRNA was used to silence its expression in the renal cancer cell lines 769-P and 786-O (**Figure 2A**). The CCK-8 assay demonstrated that IKBKE silencing significantly inhibited renal cancer cell growth (**Figure 2B**). Moreover, the number of cell colonies was dramatically reduced in the group with IKBKE knockdown compared with the control group (**Figure 2C-D**). Cell cycle analysis revealed that the depletion of IKBKE led to significant G2/M phase arrest in renal cancer cells (**Figure 2F-G**). Notably, a positive correlation was observed between IKBKE expression and Cyclin B1, as revealed by analysis of the TCGA database (**Figure 2E**), which provided a possible explanation for the G2/M phase arrest caused by IKBKE. Besides, we employed Western blotting to evaluate alterations in the cyclin B1 and cyclin A levels upon IKBKE knockout. The results revealed a decrease in cyclin B1 expression following IKBKE knockdown, whereas the cyclin A expression exhibited no significant variance (**Figure 4A**). Furthermore, Annexin V/7AAD staining and flow cytometry analysis indicated that IKBKE silencing promoted apoptosis in renal cancer cells (**Figure 2H-I**). In an *in vivo* model, subcutaneous injection of 786-O cells with IKBKE knockdown into nude mice resulted in smaller and lighter xenografts (**Figure 2J-L**). These findings collectively suggest that IKBKE silencing suppresses renal cancer cell growth both *in vitro* and *in vivo*.

### Correlation between IKBKE and sunitinib resistance in RCC

**Figure 3:**
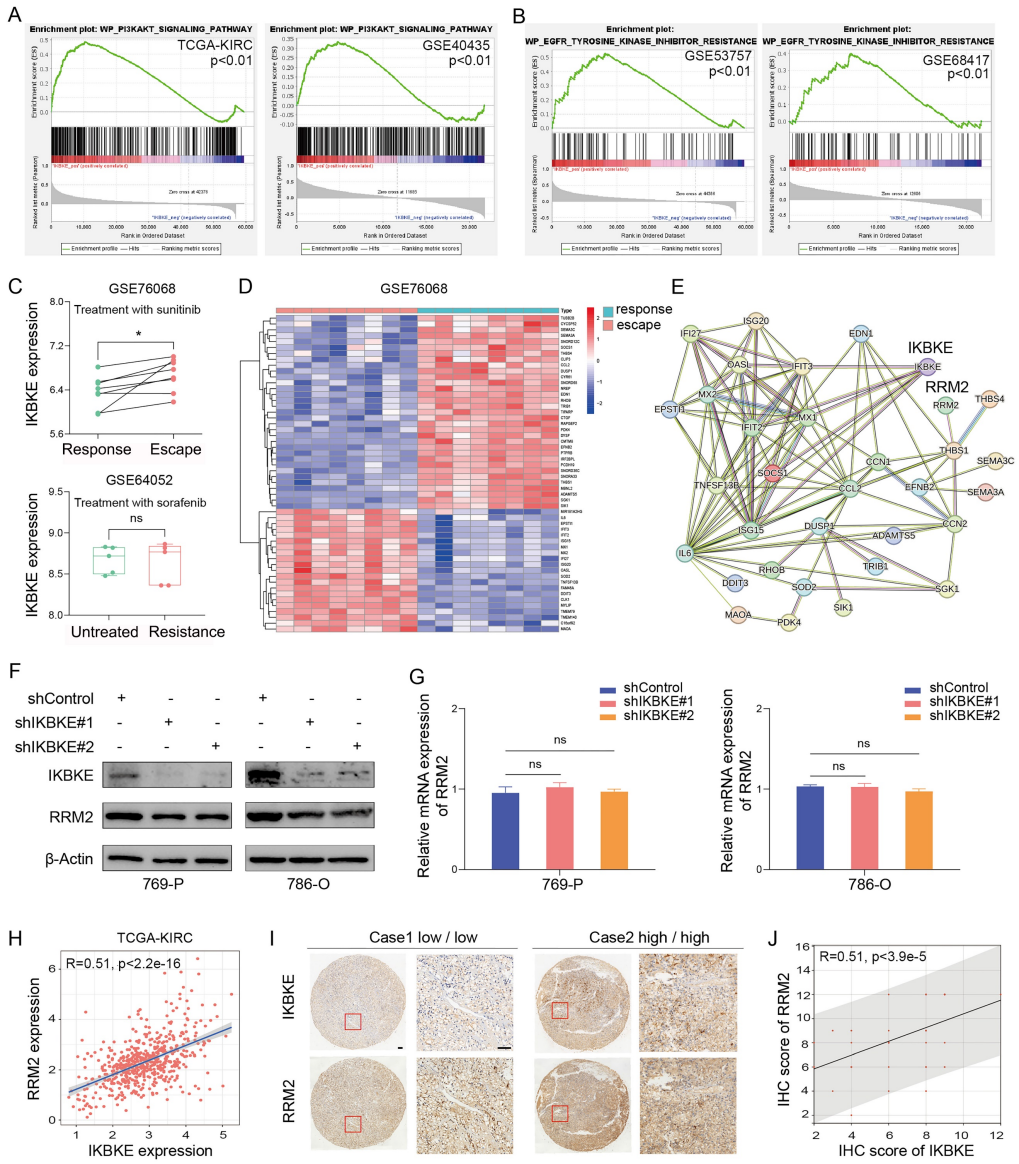
**Correlation between IKBKE and sunitinib resistance in RCC. (A)** The PI3K-AKT upregulated gene set was used to perform GSEA for the respective data sets with the IKBKE transcription level from TCGA and GSE40435 datasets. **(B)** The EGFR-Tyrosine-Kinase-Inhibitor-Resistance upregulated gene set was used to perform GSEA for the respective data sets with the IKBKE transcription level from GSE53757 and GSE68417 datasets. **(C)** IKBKE mRNA expression in sunitinib-escape and paired sunitinib-responsive samples from the GSE76068 dataset. IKBKE mRNA expression in sorafenib-resistant and untreated samples from the GSE64052 dataset. **(D)** Heatmap analysis showed the top 55 genes involved in sunitinib escape and response in the GSE76068 dataset. **(E)** PPI networks of IKBKE, RRM2, and 55 genes involved in sunitinib escape and response. **(F)** RRM2 and IKBKE protein expression levels were measured in 769-P and 786-O cells with shControl or shIKBKE by Western blotting. **(G)** RRM2 mRNA expression levels were measured in 769-P and 786-O cells with shControl or shIKBKE by qPCR. **(H)** The correlation between RRM2 expression and IKBKE expression for the data from TCGA database. **(I)** Representative immunohistochemical staining of RRM2 and IKBKE using the tissue microarray of renal cancer. Scale bars, 100 μm. **(J)** The correlation between IHC scores of RRM2 and IKBKE (*n* = 60). Each bar represents the mean values ± SD of three independent experiments except where stated. Statistical analysis was performed using Student's *t* test for (C), one-way ANOVA for (G), and Pearson's correlation analysis for (H and I). ns, no significance, *p < 0.05.

To further investigate the pathway through which IKBKE influences RCC progression, GSEA was employed to elucidate the downstream targets of IKBKE. Through the analysis of the TCGA-KIRC and GSE40435 databases, we found that high IKBKE expression resulted in significant enrichment of the PI3K-AKT signaling pathway in RCC (**Figure 3A**). Since TKIs are commonly used in RCC treatment, especially RCC with advanced stage, we verified the correlation of IKBKE with the TKI pathway. Intriguingly, we found that high IKBKE expression also resulted in remarkable enrichment of TKI resistance (**Figure 3B**). We further investigated the potential function of IKBKE in TKI resistance in RCC patients and found that patients with sunitinib resistance exhibited higher IKBKE expression (**Figure 3C up**); however, there was no significant correlation between IKBKE expression and sorafenib sensitivity in RCC patients (**Figure 3C down**).

**Figure 4:**
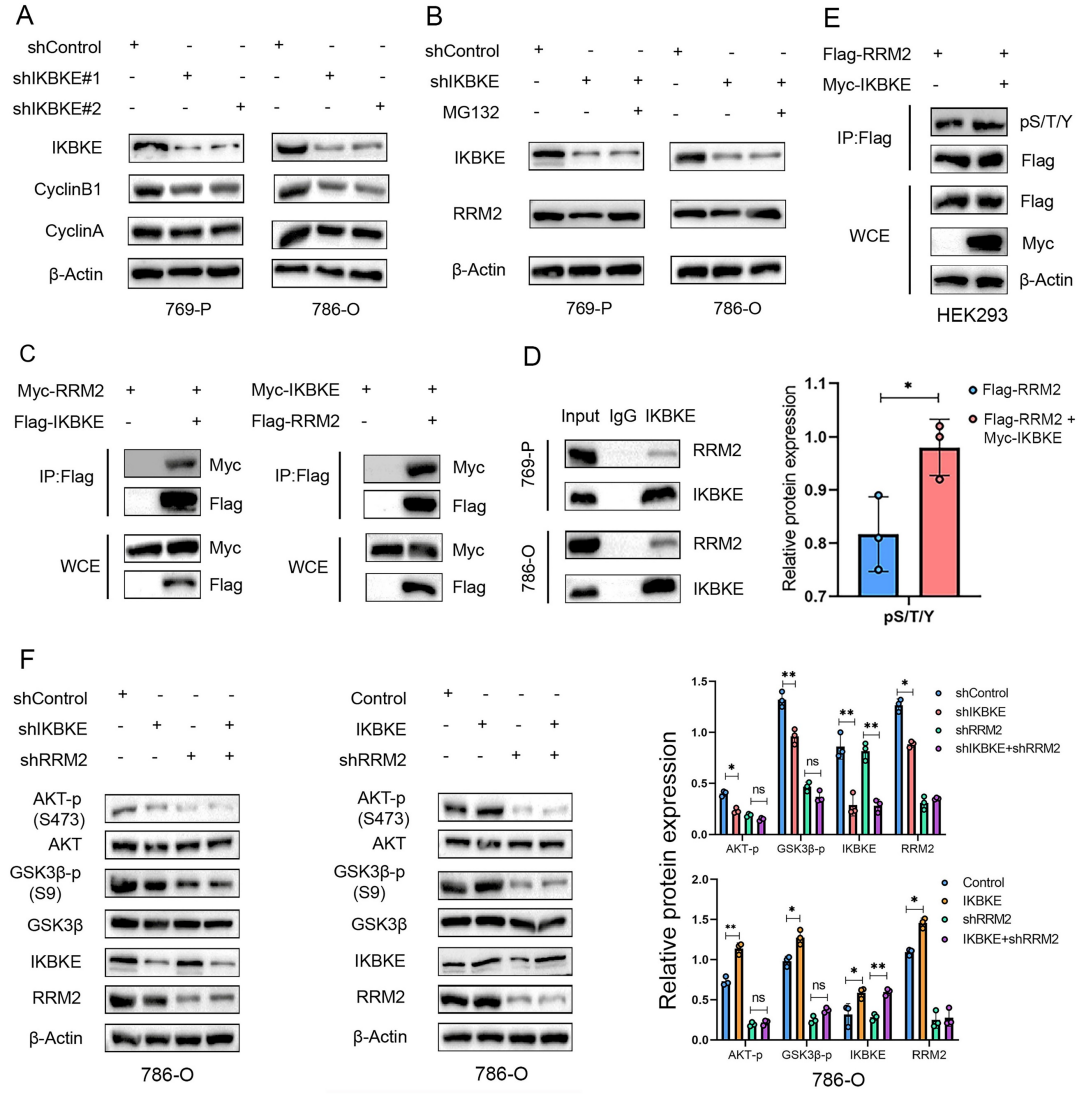
** IKBKE binds to RRM2 to activate AKT in RCC. (A)** The expression of CyclinB1 and CyclinA was determined by Western blotting in RCC cell lines treated with shIKBKE. **(B)** The expression of RRM2 was determined by Western blotting in RCC cell lines treated with shIKBKE or MG132. **(C)** Indicated plasmids were transfected into HEK293T cells and an anti-Flag antibody was used for immunoprecipitation. The precipitates were immunoblotted using Myc- and anti-Flag antibodies. **(D)** The whole-cell lysates of 769-P and 786-O cells were precipitated with IKBKE antibodies, and the precipitates were examined by immunoblotting to evaluate the endogenous interaction between IKBKE and RRM2. **(E)** RRM2-Flags were co-transfected with IKBKE plasmids into HEK293T cells, and the whole-cell lysates were subjected to immuno-affinity purification using anti-Flag magnetic beads. The expression of phospho-serine/threonine/tyrosine (p/S/T/Y) was evaluated by Western blotting. **(F)** 786-O cells were infected with the indicated lentivirus for 72 h, and subsequently, the cells were harvested for Western blotting to determine the expression of target proteins. Each bar represents the mean values ± SD of three independent experiments. Statistical analysis was performed using Student's *t* test for (E and F). ns, no significance, *p < 0.05, **p < 0.01.

Previous studies have demonstrated that the PI3K-AKT signaling pathway can facilitate cancer cell proliferation and metastasis and is closely related to sunitinib resistance in RCC 7. RRM2 is involved in the PI3K-AKT signaling pathway, and, by analyzing GSE76068 dataset, RRM2 was found to regulate the sensitivity of sunitinib by activating the PI3K-AKT pathway 14. Given our findings that IKBKE regulates RCC cell growth through the PI3K-AKT pathway and is involved in sunitinib resistance, we wondered whether there is a relationship between IKBKE and RRM2. We analyzed the sunitinib resistance dataset and identified the top 55 DEGs in patients with sunitinib- resistance (**Figure 3D**), and a protein-protein interaction (PPI) network was constructed to reveal their involvement in sunitinib resistance, with a certain correlation between IKBKE and RRM2 (**Figure 3E**). Additionally, we observed a decrease in the protein level of RRM2 when IKBKE was knocked down (**Figure 3F**), whereas the mRNA level of RRM2 remained stable (**Figure 3G**), suggesting that the regulation of RRM2 by IKBKE occurred at the posttranscriptional level. We further explored whether the loss of RRM2 protein expression induced by IKBKE depletion is reversed by MG132. Surprisingly, the proteasome inhibitor MG132 significantly diminished RRM2 downregulation induced by IKBKE silencing (**Figure 4B**). In addition, the IHC staining assays were conducted to test the protein levels of IKBKE and RRM2 in RCC patient samples from our center (FUSCC), and the results indicated that RRM2 expression exhibited a positive correlation with IKBKE expression in RCC tissues (**Figure 3I-J**). Data from the TCGA-KIRC database also confirmed the correlation between RRM2 expression and IKBKE expression (**Figure H**). Taken together, these data suggest that IKBKE regulates RRM2 protein expression and modulates sunitinib resistance in RCC.

### IKBKE binds to RRM2 to activate AKT in RCC

To further test the interaction between IKBKE and RRM2, we co-transfected Myc- and FLAG-tagged plasmids into HEK293T cells, and conducted co-immunoprecipitation (co-IP) assays with an anti-FLAG antibody. The results showed that ectopically expressed IKBKE significantly interacted with RRM2, and vice versa (**Figure 4C**). The interaction between endogenous IKBKE and RRM2 was confirmed in 769-P and 786-O cells (**Figure 4D**). IKBKE is a kinase; therefore, it is necessary to verify whether IKBKE regulates the phosphorylation of RRM2.

In the current study, we evaluated the pan-phosphorylation level of RRM2 after manipulating IKBKE expression in HEK293T cells and found that the pan-phosphorylation level of RRM2 was upregulated when IKBKE overexpression (**Figure 4E**). Considering the role of RRM2 in the activation of AKT pathway 14 and our findings that high IKBKE expression resulted in the significant enrichment of the PI3K-AKT signaling pathway, we further conducted Western blotting to determine whether RRM2 depletion has an effect on the AKT signaling caused by IKBKE depletion or overexpression. The Western blotting results indicated that IKBKE knockdown could not further decrease the phosphorylation levels of AKT and GSK3β in renal cancer cells in the context of RRM2 silencing (**Figure 4F**, left panel), and importantly RRM2 silencing attenuated the ability of IKBKE overexpression to alter the phosphorylation levels of AKT and GSK3β in renal cancer cells (**Figure 4F**, right panel). Collectively, these findings indicate that IKBKE physically interacts with and phosphorylates RRM2 to activate the AKT signaling pathway in renal cancer.

### IKBKE promotes RCC progression by upregulating RRM2

**Figure 5:**
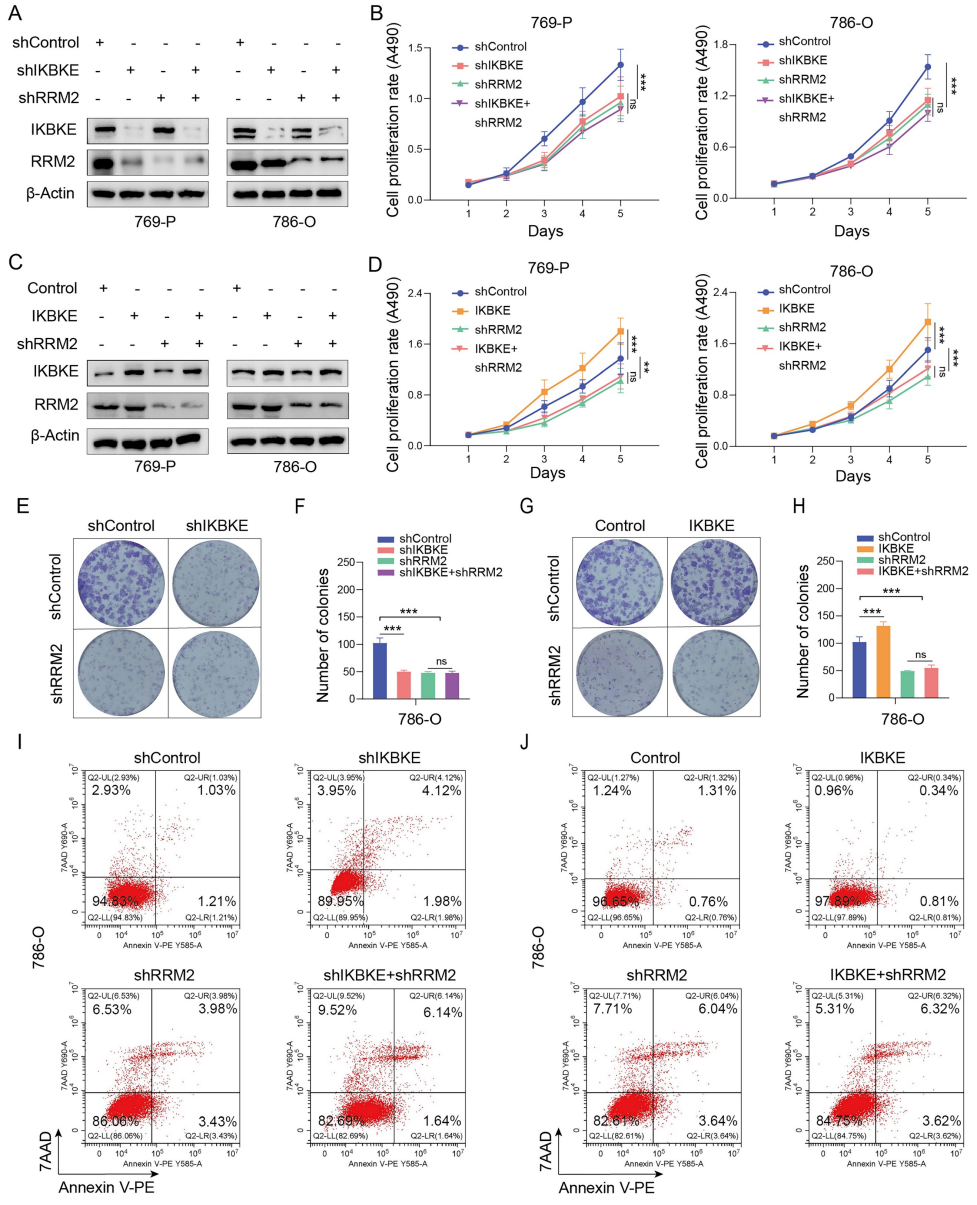
**IKBKE promotes RCC progression by upregulating RRM2. (A)** The expression of IKBKE and RRM2 was measured in RCC cell lines treated with shIKBKE or shRRM2 by Western blotting. **(B)** The cell proliferation rate of RCC cell lines infected with the indicated lentivirus was evaluated through CCK-8 assays. **(C)** The expression of IKBKE and RRM2 was measured in RCC cell lines treated with IKBKE or shRRM2 by Western blotting. **(D)** The cell proliferation rate of RCC cell lines infected with the indicated lentivirus was evaluated through CCK-8 assays. **(E-H)** The colony numbers of RCC cell lines infected with the indicated lentivirus were evaluated through colony formation assays. The diameter of each well in panel C is approximately 35 mm. **(I-J)** The cell apoptosis of RCC cell lines infected with the indicated lentivirus was evaluated by Annexin V-7AAD flow cytometry. Each bar represents the mean values ± SD of three independent experiments. Statistical analysis was performed using one-way ANOVA for (B, D, F, and H). ns, no, significance, **p < 0.01, ***p < 0.001.

Given that IKBKE regulates RRM2 protein expression in RCC, we wondered whether IKBKE influences RCC progression via an RRM2-mediated pathway. To this end, we first constructed stable RCC cell lines with IKBKE or RRM2 knockdown alone or a double knockdown of both IKBKE and RRM2 (**Figure 5A**). CCK-8 assays indicated that knockdown of either IKBKE or RRM2 markedly reduced the growth of 769-P and 786-O cells. Importantly, IKBKE knockdown could not further decrease the proliferation of renal cancer cells in the context of RRM2 silencing (**Figure 5B**). Colony formation and Annexin V/7AAD staining assays demonstrated that RRM2 silencing could abolish the damaging effects of IKBKE on renal cancer cell growth and apoptosis (**Figure 5E-F, 6I**). Subsequently, we constructed stable renal cancer cells with RRM2 knockdown and IKBKE overexpression (**Figure 5C**) and found that the effect of IKBKE overexpression on cell proliferation and apoptosis was diminished in shRRM2 cells (**Figure 5D, G, H, J**). These findings reveal that IKBKE regulates the progression of renal cancer cells via an RRM2-mediated pathway.

### The IKBKE-RRM2 axis regulates sunitinib sensitivity in RCC

**Figure 6:**
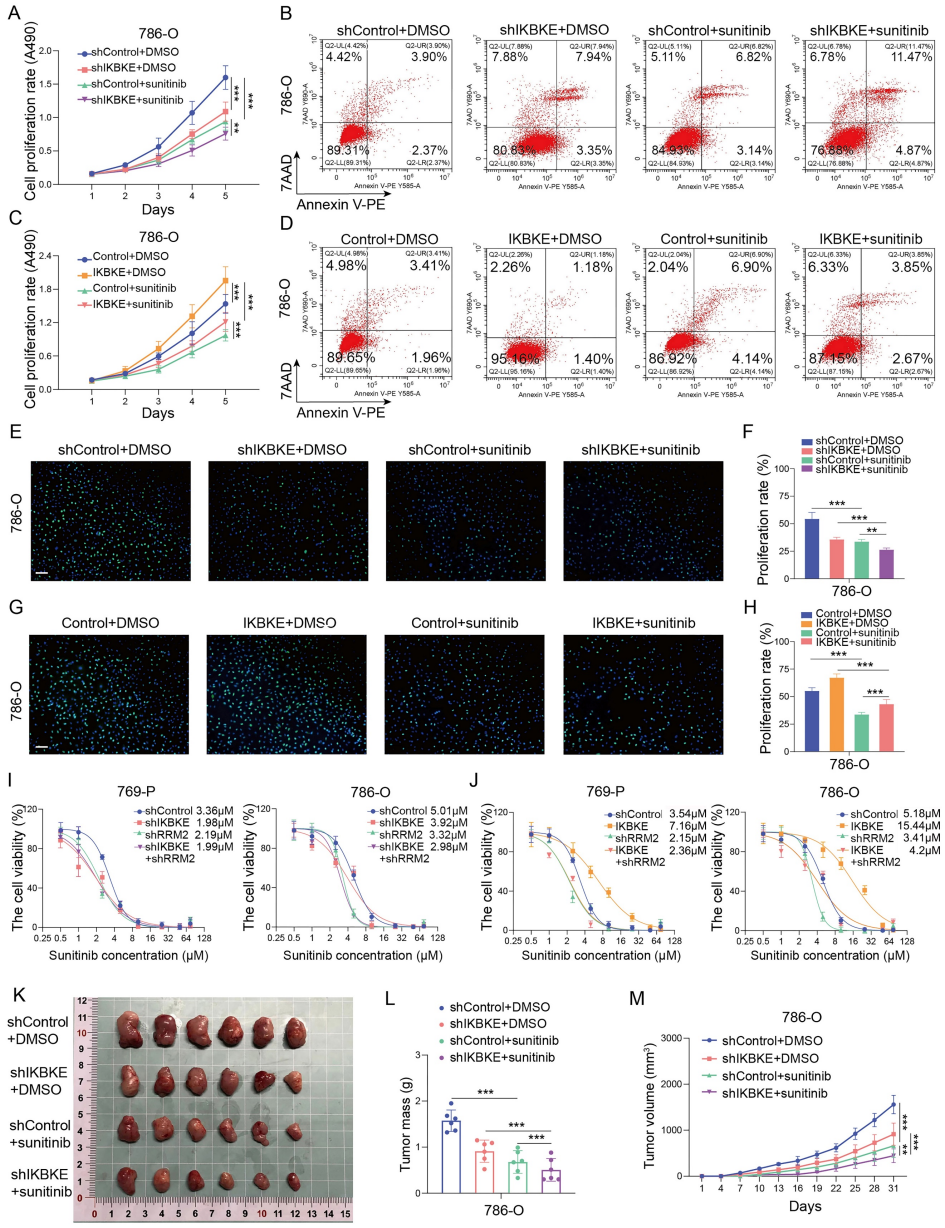
**The IKBKE-RRM2 axis regulates sunitinib sensitivity in RCC. (A-H)** 786-O cells were infected with the indicated lentivirus for 72 h, and subsequently, the cells were treated with or without sunitinib (2 µM). The cell proliferation rate was evaluated through CCK-8 (A, C) and EDU (E and G, scale bars, 50 μm) assays, and cell apoptosis was evaluated by Annexin V-7AAD flow cytometry (B, D). **(I-J)** 769-P and 786-O cells were infected with the indicated lentivirus for 72 h, and subsequently, the cells were treated with a series of concentrations of sunitinib for 48 h. Cell viability was determined by a CCK-8 assay. **(K-M)** 786-O cells were infected with the indicated lentivirus for 72 h. After puromycin (5 μg/mL) selection, cells were collected and subcutaneously injected into the nude mice (*n* = 6 mice per group). These mice were treated with or without sunitinib (20 mg/kg). The xenografts in each group are shown in Panel K, the tumor mass is shown in Panel L, and the tumor growth curve is shown in Panel M. Each bar represents the mean values ± SD of three independent experiments except where stated. Statistical analysis was performed using one-way ANOVA for (A, C, F, H, L, and M). *p < 0.05, **p < 0.01, ***p < 0.001.

Given that IKBKE is associated with sunitinib sensitivity in RCC, we investigated whether this regulation is mediated by RRM2. Previous studies have shown that RRM2 can modulate sunitinib sensitivity 14. The CCK-8 assay, EDU assay, and Annexin V/7AAD staining assay revealed that silencing IKBKE increased sunitinib sensitivity in renal cancer cells (**Figure 6A-B, 6E-F**), while overexpression of IKBKE decreased the sensitivity of renal cancer cells to sunitinib (**Figure 6C-D, 6G-H**). Furthermore, we found that RRM2 depletion mitigated the alterations in the IC50 values of sunitinib induced by either the overexpression or knockdown of IKBKE in renal cancer cells (**Figure 6I-J**). We further carried out angiogenesis experiments to prove the effect of IKBKE/RRM2 on sunitinib sensitivity and the results showed that inhibition of IKBKE expression reduced the angiogenic capability, whereas overexpression of IKBKE promoted angiogenesis. Importantly, IKBKE knockdown could not further decrease the angiogenesis of renal cancer cells in the context of RRM2 silencing, and RRM2 silencing attenuated the effect of IKBKE overexpression on angiogenesis (Supplementary **Figure S1A-D**). These findings were consistent with our data showing that IKBKE regulated RCC progression through RRM2. Subsequent *in vivo* assays confirmed the *in vitro* study result that IKBKE modulated sunitinib sensitivity in renal cancer cells (**Figure 6K-M**). Taken together, these data demonstrate that IKBKE can regulate the sensitivity of renal cancer cells to sunitinib, and such regulation is primarily through the IKBKE-RRM2 axis.

### An IKBKE inhibitor represses cell growth and enhances sunitinib sensitivity in RCC

**Figure 7:**
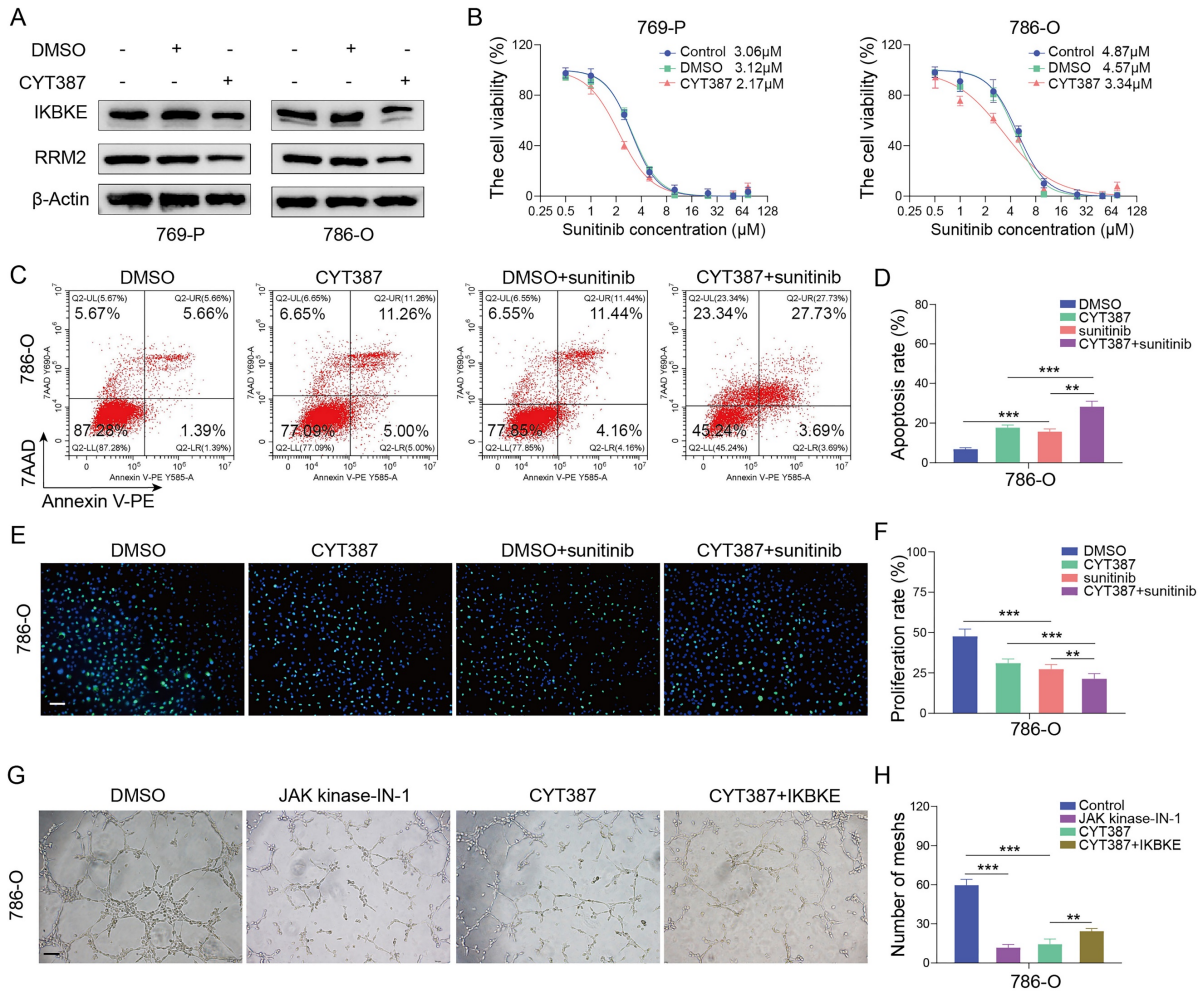
**The IKBKE inhibitor CYT387 regulates sunitinib sensitivity in RCC. (A)** 769-P and 786-O cells were treated with or without 5 μM CYT387 for 48 h. The protein expression of IKBKE and RRM2 was detected by Western blotting. **(B)** 769-P and 786-O cells were treated with control, DMSO or CYT387 (5 μM) for 48 h; subsequently, these cells were harvested and treated with a series of doses of sunitinib for 48 h. Cell viability was determined by CCK-8 assays. **(C-F)** 786-O cells were treated with DMSO or CYT387 for 48 h. Subsequently, these cells were treated with sunitinib (2 µM). Cell apoptosis was evaluated by Annexin V-7AAD flow cytometry (C, D). The cell proliferation rate was evaluated by EDU assays (E, F). Scale bar = 50 μm. **(G-H)** 786-O cells were treated with indicated constructs. After 48 h, the cell medium was collected for *in vitro* angiogenesis assays. Scale bar = 100 μm. Each bar represents the mean values ± SD of three independent experiments. Statistical analysis was performed using one-way ANOVA for (D, F, and H). **p < 0.01, ***p < 0.001.

We have shown that IKBKE can not only promote RCC cell growth but also modulate sunitinib sensitivity. However, it remains unclear whether pharmacological inhibition of IKBKE can affect renal cancer cells. CYT387, an IKBKE inhibitor, was utilized to investigate the effect of IKBKE pharmacological inhibition on sunitinib sensitivity. Hence, renal cancer cells were treated with a gradient concentration of CYT387 (ranging from 0.5 μM to 64 μM) to measure the IC50 using CCK-8 assays (**Figure S2A**). CYT387 treatment decreased the expression levels of both IKBKE and RRM2 in renal cancer cells (**Figure 7A**), and 5 μM CYT387 markedly inhibited renal cancer cell growth (**Figure S2B**). Additionally, colony formation counts dramatically decreased in the CYT387 treatment group compared with the control group (**Figure S2C-D**). Cell cycle analysis demonstrated that CYT387 treatment induced G2/M phase arrest in renal cancer cells (**Figure S2E-F**) (*p* <0.05). *In vivo*, we observed that xenografts in the CYT387-treated group were markedly lighter and smaller than those in the control group (**Figure S2G-I**).

As the downregulation of IKBKE can enhance sunitinib sensitivity in renal cancer cells, we evaluated whether CYT387 could promote sunitinib sensitivity in renal cancer cells. Notably, CYT387 treatment reduced the IC50 values of sunitinib in both 769-P and 786-O cells (**Figure 7B**). Furthermore, the Annexin V/7AAD staining assay and EDU assay indicated that CYT387 treatment enhanced the sensitivity of renal cancer cells to sunitinib (**Figure 7C-F**). CYT387, while primarily used as an IKBKE inhibitor, also inhibits JAK kinases. By angiogenesis assays, we found that both CYT387 and JAK kinase inhibitors significantly inhibited the angiogenesis of HUVEC cells. Importantly, on the basis of CYT387 treatment, replenishing IKBKE expression could promote the angiogenesis of HUVEC cells compared with the CYT387 treatment (**Figure 7G-H**). Taken together, our data suggest that the IKBKE inhibitor CYT387 can repress RCC cell growth and enhance sunitinib sensitivity, indicating that such drugs have the potential to treat RCC patients, especially RCC patients who are resistant to sunitinib.

## Discussion

Emerging evidence supports the importance of systemic therapies, such as targeted therapy or immunotherapy, as indispensable interventions for advanced RCC 15, 16. TKIs function by inhibiting the kinase activity of members of the vascular endothelial growth factor receptor (VEGFR) family and platelet-derived growth factor receptor (PDGFR) family. Notably, sunitinib, a prominent TKI, exerts significant inhibitory effects on tumor growth by diminishing tumor angiogenesis 17, 18. However, cancer cells, such as prostate and breast cancer cells, can develop resistance to initially effective drugs 19-22. Notably, sunitinib resistance frequently occurs in RCC patients after approximately one year of treatment 23. Consequently, there is an imperative need to investigate the potential mechanisms underlying sunitinib resistance, as well as to identify predictive biomarkers for further overcoming sunitinib resistance.

In the current study, we found that IKKBE was upregulated in various cancers, including RCC, through pancancer analysis. Remarkably, IKBKE expression displayed a robust positive correlation with clinical staging. Increasing evidence suggests that IKBKE is overexpressed in numerous malignancies 24-26, and its overexpression has been correlated with the proliferation, invasion, and metastasis of various cancers 27-29. Furthermore, IKBKE has been shown to activate the AKT pathway, which plays an important role in tumor progression and chemotherapy resistance 30-32. Intriguingly, we found that IKBKE silencing effectively suppressed renal cancer cell growth and stimulated cell apoptosis. In addition, our analysis of several RCC datasets indicated a close association between IKBKE expression and the AKT pathway. Furthermore, we observed upregulated IKBKE expression in samples from sunitinib-resistant RCC patients. Subsequently, we confirmed a positive correlation between IKBKE and RRM2, and that these two factors can regulate sunitinib sensitivity by activating the AKT pathway in RCC 14. Consequently, we hypothesized that IKBKE promotes the RCC progression and sunitinib resistance by regulating RRM2.

RRM2 functions as a ribonucleotide reductase subunit that can catalyze synthesis, and its aberrant upregulation was reported to be correlated with enhanced cell proliferation and chemotherapy resistance in various tumors 33, 34. Notably, miR-99a-3p, a microRNA associated with RRM2, was downregulated in sunitinib-resistant RCC cells, indicating potential RRM2 overexpression in sunitinib-resistant RCC cells 35. RRM2 overexpression can promote malignant peripheral nerve sheath tumor cell proliferation by activating the AKT pathway 36. These findings suggest a potential role for RRM2 in regulating RCC progression via the AKT pathway. In the current study, we demonstrated that IKBKE could regulate RRM2 expression at the posttranscriptional level. Notably, altering IKBKE expression did not further influence cell growth or sunitinib sensitivity when RRM2 was knocked down in renal cancer cells, demonstrating that IKBKE primarily modulates cell growth and sunitinib sensitivity through the RRM2-mediated AKT pathway.

CYT387, identified as an IKBKE inhibitor, has been shown to inhibit the proliferation, migration, and invasion of glioma cells while inducing cell cycle arrest at the G2/M checkpoint 37. Consistently, we found that CYT387 treatment resulted in the downregulated expression of both IKBKE and RRM2, and CYT387 effectively inhibited renal cancer cell proliferation and arrested the cell cycle at the G2/M checkpoint. Furthermore, CYT387 decreased the IC50 value of sunitinib in renal cancer cells. To our knowledge, this study is the first to demonstrate that the downregulation of IKBKE and the use of its inhibitor, CYT387, could suppress the growth of RCC cells and enhance their sensitivity to sunitinib.

Although we explicitly elucidated that IKBKE induces RCC progression and sunitinib resistance through the RRM2-AKT pathway, which can be suppressed by CYT387, an IKBKE inhibitor, there are still a few issues to be studied. First, the underlying mechanism by which IKBKE regulates RRM2 expression remains to be investigated. Second, whether IKBKE induces RCC progression and sunitinib resistance by regulating the AKT signaling pathway via alternative targets or affecting factors other than RRM2, such as NF-κB 24, EGFR 38, or even the immune microenvironment 39, deserves further exploration. Finally, potential challenges in translating CYT387's efficacy from preclinical models to clinical applications, such as the variability in patient responses, potential off-target effects, and the long-term safety profile of CYT387, should be fully evaluated. Additionally, also CYT387's optimal dosing regimens, combination therapies, and both efficacy and safety in diverse patient populations need to be further studied.

## Conclusion

**Figure 8:**
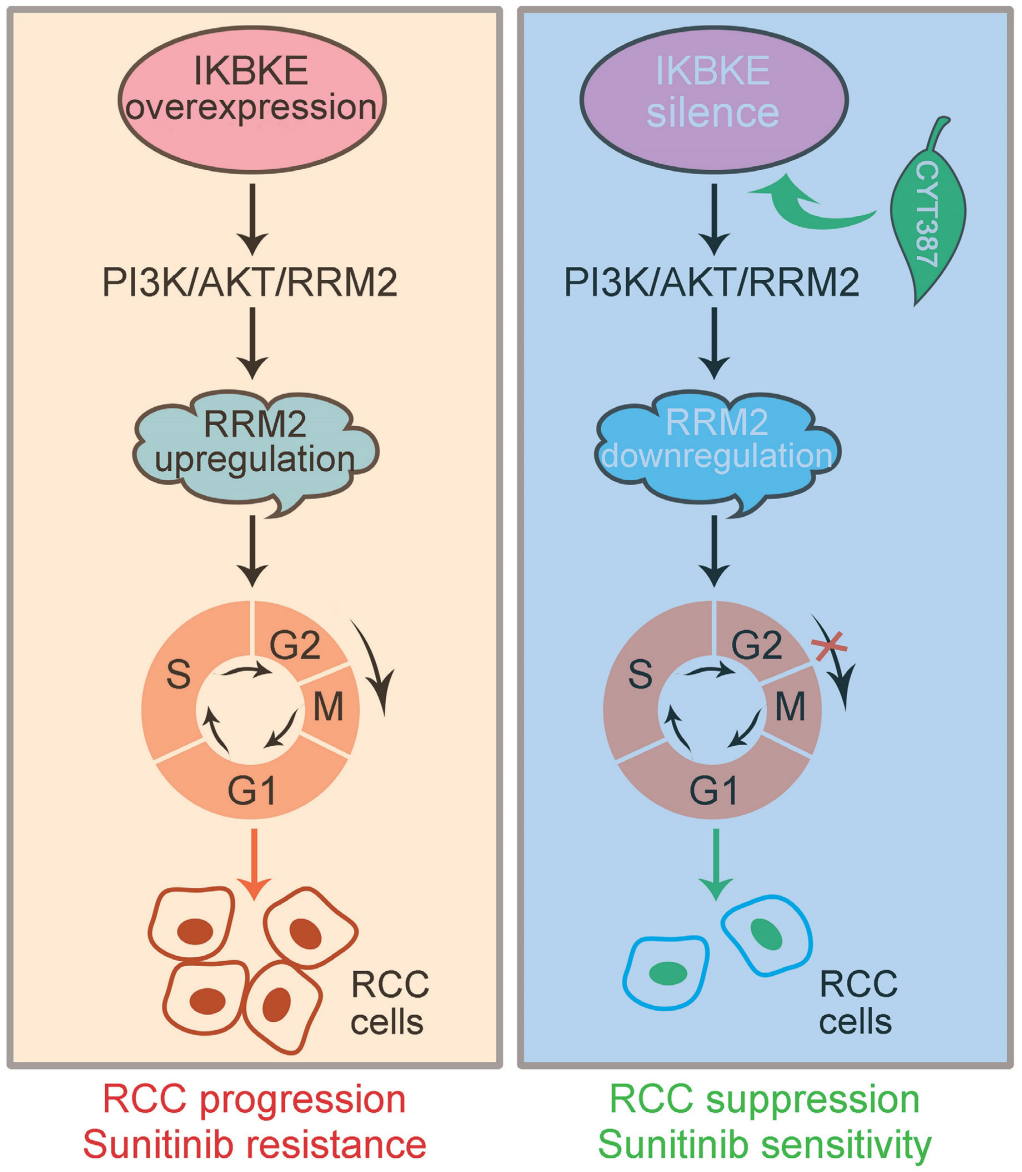
**A working model of the IKBKE-RRM2 axis regulating RCC progression and sunitinib resistance. (Left)** IKBKE overexpression induces RCC progression and sunitinib resistance by upregulating RRM2 through the RRM2-mediated PI3K/AKT pathway. **(Right)** IKBKE silencing downregulates RRM2 and induces cell cycle arrest at G2/M phase through the RRM2-mediated PI3K/AKT pathway, suppressing the progression of renal cancer cells and enhancing sunitinib sensitivity. In addition, the IKBKE inhibitor CYT387 can inhibit IKBKE expression and restore sunitinib sensitivity in RCC cells.

In conclusion, IKBKE induces RCC progression and sunitinib resistance through the RMM2-mediated AKT signaling pathway. Silencing IKBKE downregulates RMM2 expression to restrain RCC cell growth and enhance sunitinib sensitivity. These effects can be achieved by the IKBKE inhibitor CYT387 (**Figure 8**), suggesting that the IKBKE-RRM2 axis plays a pivotal role in regulating RCC progression and sunitinib resistance and that IKBKE may be a potential target for the treatment of RCC.

## Supplementary Material

Supplementary figures and tables.

## Abbreviations

AKT: protein kinase B
CCK8: cell counting kit-8
DEGs: differentially expressed genes
EGFR: epidermal growth factor receptor
GEO: gene expression omnibus
GSEA: gene set enrichment analysis
IC50: 50% maximal inhibitory concentration
HUVEC: human umbilical vein endothelial cell
IHC: immunohistochemistry
IKBKE: inhibitor of nuclear factor kappa B kinase subunit epsilon
IP: immunoprecipitation
KIRC: kidney renal clear cell carcinoma
NES: normalized enrichment score
OS: overall survival
PDGFR: platelet-derived growth factor receptor
PFS: progression-free survival
PI: propidium iodide
PPI: protein-protein interaction
RCC: renal cell carcinoma
ROC: receiver operating characteristic curve
RRM2: ribonucleotide reductase M2
TCGA: The Cancer Genome Atlas
TKIs: tyrosine kinase inhibitors
TMA: tissue microarrays
VEGFR: vascular endothelial growth factor receptor
