# Supplementary material for: IKBKE regulates renal cell carcinoma progression and sunitinib resistance through the RRM2-AKT pathway
Source: Int J Biol Sci. 2024 Nov 11;20(15):6146–61. doi: 10.7150/ijbs.102666 (PMC11628342; doi:10.7150/ijbs.102666)
Supplement: Supplementary file 1 — Supplementary figures and tables. [file ijbsv20p6146s1.pdf]

Supplementary Materials

Figure S1. RRM2 silencing attenuates the ability of IKBKE knockdown or overexpression to affect angiogenesis. (**A–D**) 786-O cells were infected with the indicated lentivirus for 72 h, and subsequently, the cell medium was collected for in vitro angiogenesis assays. Scale bar = 100 μm. Statistical analysis was performed using one-way ANOVA for (B and D). ns, no significance; ***$p < 0.001$

Figure S2. The IKBKE inhibitor CYT387 inhibits cell growth in RCC. (**A**) The viability of 769-P and 786-O cells treated with CYT387 for 24 h, 48 h, and 72 h, respectively was measured through CCK-8 assays. (**B**) Proliferation rates of 769-P and 786-O cells in the control, DMSO, and CYT387 (5 μM) groups were compared through CCK-8 assays. (**C–D**) Colony numbers of 769-P and 786-O cells in the control, DMSO, and CYT387 (5 μM) groups were compared through colony formation assays. The diameter of each well in panel C is approximately 35 mm. (**E–F**) Cell cycle analysis of 769-P and 786-O cells in the control, DMSO, and CYT387 (5 μM) groups was determined through flow cytometry. (**G–I**) 786-O cells were subcutaneously injected into nude mice for the xenograft assay (n = 6 mice/group). When the volume of xenografts reached 50 mm^3, mice were randomly divided into two groups and treated with CYT387 (20 mg/kg, intraperitoneal injection with a volume of 200 μL for every 3 days) or constant volume solvents (DMSO), respectively. Xenografts in each group were photographed in panel G, the tumor mass was demonstrated in panel H, and the tumor growth curve was indicated in panel I. Statistical analysis was performed using one-way ANOVA for (D) and Student’s *t* test for (H and I). ***$p < 0.001$

Figure S1

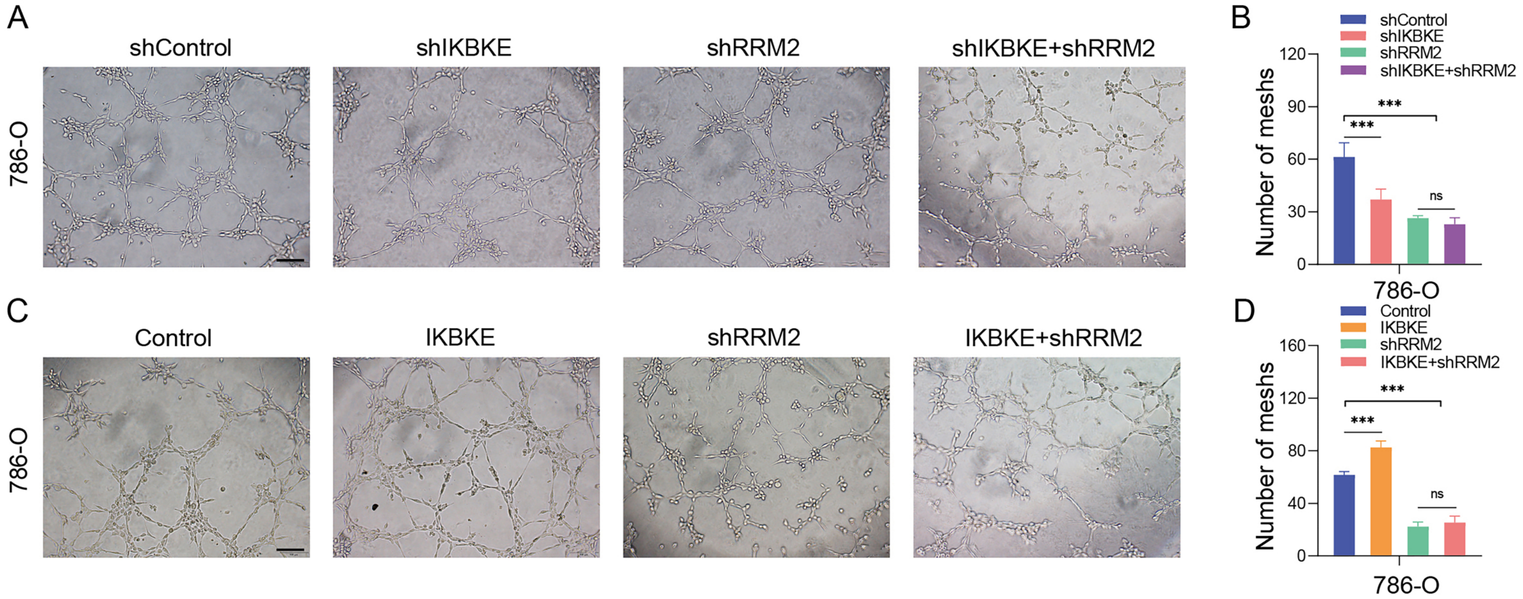
A
shControl
shIKBKE
shRRM2
shIKBKE+shRRM2
786-O
B
shControl
shIKBKE
shRRM2
shIKBKE+shRRM2
Number of meshs
120
90
60
30
0
***
***
ns
786-O
C
Control
IKBKE
shRRM2
IKBKE+shRRM2
786-O
D
Control
IKBKE
shRRM2
IKBKE+shRRM2
Number of meshs
160
120
80
40
0
***
***
ns
786-O

figure S2

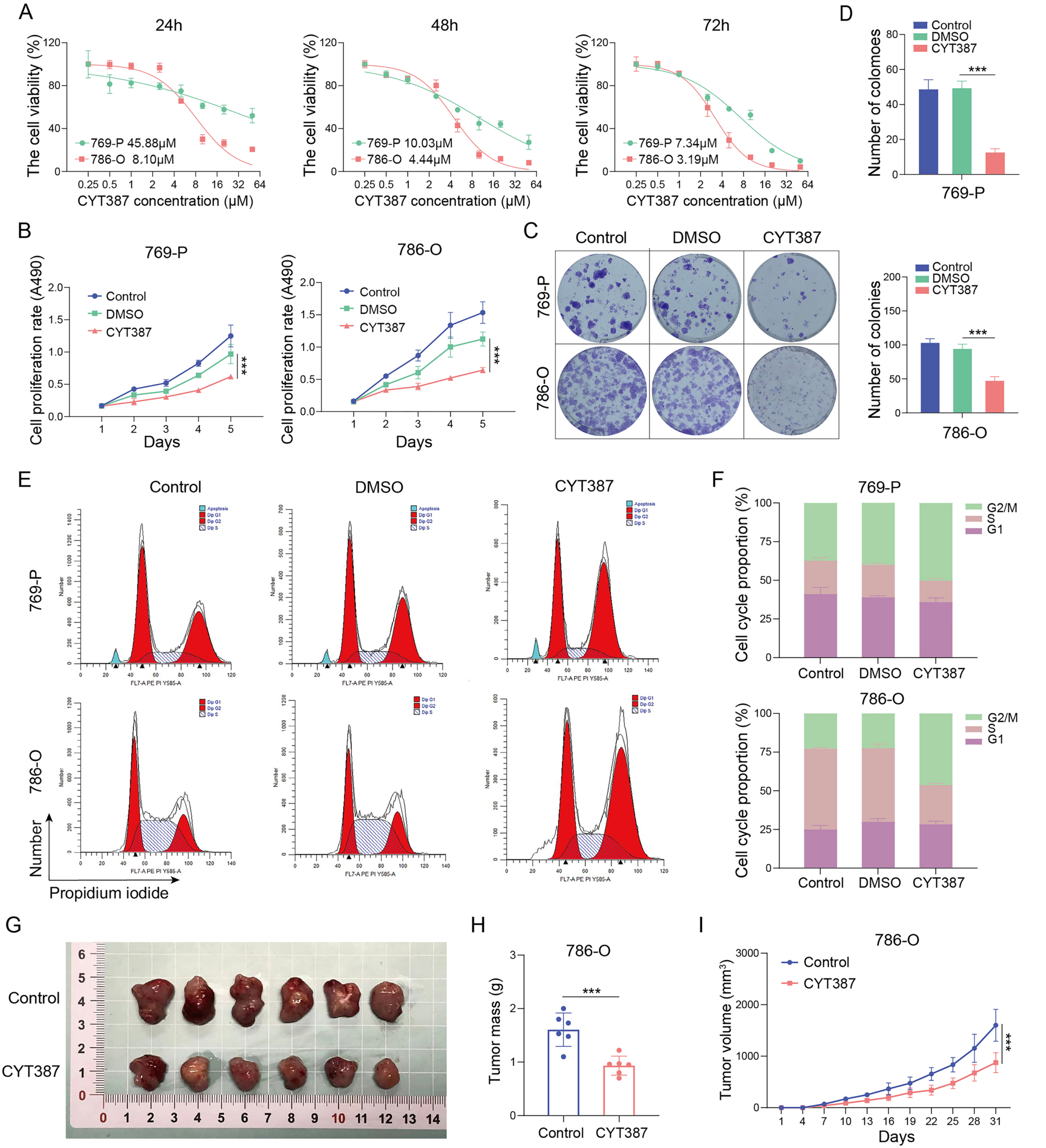

A
24h
48h
72h
The cell viability (%)
769-P 45.88μM
786-O 8.10μM
769-P 10.03μM
786-O 4.44μM
769-P 7.34μM
786-O 3.19μM
CYT387 concentration (μM)
B
769-P
786-O
Cell proliferation rate (A490)
Control
DMSO
CYT387
Days
C
Control
DMSO
CYT387
769-P
786-O
Number of colonies
D
Number of colomoes
769-P
786-O
E
Control
DMSO
CYT387
769-P
786-O
Number
Propidium iodide
F
769-P
786-O
Cell cycle proportion (%)
G2/M
S
G1
Control
DMSO
CYT387
G
Control
CYT387
H
786-O
Tumor mass (g)
Control
CYT387
I
786-O
Tumor volume (mm³)
Control
CYT387
Days
